# KIF21B, Ubiquitinated by TRIM3, Exerts Oncogenic Role in T-Cell Acute Lymphoblastic Leukemia by Activating Wnt/β-Catenin Pathway

**DOI:** 10.3390/cancers18091327

**Published:** 2026-04-22

**Authors:** Yu Sun, Yuhao Xu, Chao Lu

**Affiliations:** Department of Pediatrics, The First Affiliated Hospital with Nanjing Medical University, Nanjing 210029, China; victmsun@163.com (Y.S.); 15250962215@163.com (Y.X.)

**Keywords:** KIF21B, acute lymphoblastic leukemia, ubiquitination, Wnt/β-catenin signaling, prognosis

## Abstract

Background: Pediatric T-cell acute lymphoblastic leukemia (T-ALL) remains a therapeutic challenge, underscoring an urgent need for novel therapeutic targets. Although the Kinesin family member KIF21B has been implicated in solid tumors, its specific role and regulatory mechanisms in T-ALL are poorly understood. This study aimed to investigate the clinical significance, biological functions, and upstream regulatory mechanisms of KIF21B in T-ALL. Methods: We analyzed public databases to assess KIF21B expression levels and their correlation with patient prognosis. In vitro experiments including KIF21B knockdown were performed to evaluate its effects on leukemia cell proliferation, apoptosis, and mitotic progression. In vivo studies using a mouse model were conducted to examine the impact of KIF21B depletion on leukemia progression. Mechanistic investigations focused on the Wnt/β-catenin signaling pathway and the role of TRIM3 as a potential upstream regulator. Results: Public database analysis revealed that KIF21B is significantly overexpressed in T-ALL patients, and elevated KIF21B levels strongly predict poor prognosis. In vitro experiments demonstrated that KIF21B knockdown inhibited proliferation, induced apoptosis, and caused severe mitotic defects. These findings were further supported by in vivo evidence showing that KIF21B depletion markedly suppressed leukemia progression in mice. Mechanistically, we proved that KIF21B activates the Wnt/β-catenin signaling pathway and identified TRIM3 as a novel E3 ubiquitin ligase that acts as an upstream negative regulator by promoting KIF21B ubiquitination and degradation. Conclusions: Our results define KIF21B as a key oncoprotein in T-ALL and unveil a previously unrecognized TRIM3–KIF21B–β-catenin regulatory axis, highlighting its potential as a promising therapeutic strategy for this disease.

## Simple Summary

Pediatric T-cell acute lymphoblastic leukemia (T-ALL) is a challenging type of blood cancer, and new treatment targets are urgently needed. This study explored the role of a protein called KIF21B in this disease. We found that KIF21B is present at high levels in patients with T-ALL and is linked to a poorer outlook. Experiments showed that reducing KIF21B in leukemia cells slowed their growth and caused them to die. Further work revealed that KIF21B works by activating a key signaling pathway and that it is normally kept in check by another protein, TRIM3, which marks KIF21B for destruction. Our findings identify KIF21B as a key driver of T-ALL and suggest that targeting it, or its regulatory pathway, could offer a promising new approach for treating this aggressive cancer.

## 1. Introduction

Pediatric T-cell acute lymphoblastic leukemia (T-ALL) remains a therapeutic challenge, with approximately 20% of patients experiencing relapse due to a limited understanding of molecular drivers [1,2]. While genomic studies have identified recurrent mutations in NOTCH1 and PTEN [3,4], a significant portion of T-ALL cases lack these canonical lesions, suggesting the involvement of other oncogenic mechanisms [5]. Emerging evidence suggests that cytoskeletal regulators may represent a novel class of therapeutic targets [6,7,8]. Among these, kinesin superfamily proteins (KIFs) have been implicated in malignancy through their roles in intracellular transport and cell division [9,10,11]. Notably, the microtubule-associated motor protein KIF21B has drawn attention due to its reported oncogenic functions in solid tumors [12,13] and critical roles in neural development [14]. Intriguingly, beyond its structural role, the cytoskeleton is increasingly recognized as a dynamic signaling hub that can influence key oncogenic pathways, including those critical for leukemia cell survival and proliferation [15,16].

The regulation of kinesin proteins in cancer, particularly in hematological malignancies, is poorly understood. Post-translational modifications, such as ubiquitination, are key mechanisms controlling protein stability, localization and activity of oncogenic drivers [17]. The TRIM (Tripartite Motif) family of E3 ubiquitin ligases has emerged as critical regulators of diverse cellular processes and are frequently dysregulated in cancer [18,19]. In neurons, TRIM3 has been shown to post-translationally regulate KIF21B [20], yet whether this regulatory axis exists or plays any role in cancer biology remains completely unexplored [20]. Our preliminary data identified aberrant overexpression of KIF21B in primary T-ALL samples, which correlated with poor patient outcomes, suggesting a potential functional importance in leukemogenesis.

Therefore, significant gaps exist in our knowledge. The functional role and mechanistic contribution of KIF21B in T-ALL are unknown. The upstream regulators controlling KIF21B in any cancer context are elusive. The way that KIF21B integrates with established oncogenic signaling pathways in leukemia is unclear. Based on this background, this study was designed to investigate the oncogenic role of KIF21B in T-ALL. We aimed to determine its clinical relevance, elucidate its biological functions and identify both its upstream regulator and key downstream effector pathway. Our findings bridge the discrete fields of cytoskeletal biology, ubiquitin signaling and leukemia research, potentially offering new perspectives for targeting high-risk pediatric T-ALL.

## 2. Materials and Methods

### 2.1. Data Acquisition and Processing

We downloaded STAR-counts data and corresponding clinical information for pediatric T-ALL from the TARGET database. We then extracted data in TPM format and performed normalization using the log2(TPM + 1) transformation. After retaining samples that included both RNAseq data and clinical information, we ultimately performed further analysis. We used the log rank test to compare the survival differences between the two groups mentioned above in the KM survival analysis. All analytical methods and R packages were performed using R software version v4.0.3 (The R Foundation for Statistical Computing, 2020), with *p*  <  0.05 being considered as statistically significant.

### 2.2. Collection and Processing of PBMCs from T-ALL Patients

Peripheral blood samples from patients with T-ALL and healthy controls were obtained from the Department of Pediatrics, Jiangsu Provincial People’s Hospital, from September 2023 to June 2025. Clinical and laboratory data were retrospectively collected from medical records. The detailed demographic, clinical and molecular characteristics of the T-ALL cohort are summarized in Table S1. Peripheral venous blood was collected using standard anticoagulant tubes and processed within 2 h to preserve cell viability. Using low-speed centrifugation, red blood cells and granulocytes were sedimented, while mononuclear cells (PBMCs) accumulated at the buffy coat layer. This whitish interphase was carefully aspirated, washed twice gently with physiological buffer to remove residual plasma and separation medium, and quickly assessed under a microscope with dye exclusion to ensure >95% cell viability. Cells were resuspended in serum-containing medium for subsequent experiments.

### 2.3. RNA Extraction and qRT-PCR

When suspended cells reached a density of approximately 8 × 10^5^ cells/mL, the culture medium was collected and centrifuged at 200× *g* for 5 min. The pellet was washed with pre-cooled PBS and lysed in Trizol reagent. After adding chloroform (0.2 mL per 1 mL Trizol), the mixture was vigorously shaken, incubated on ice for 3 min and centrifuged at 4 °C and 12,000 rpm for 15 min. The upper aqueous phase was collected, mixed with an equal volume of isopropanol to precipitate RNA, washed with ethanol and centrifuged again. The RNA pellet was air-dried and dissolved in DEPC water. Concentration was measured using NanoDrop, Wilmington, DE, USA. cDNA was synthesized using HiScript Q RT SuperMix (Vazyme, Nanjing, China). qRT-PCR was performed using a real-time PCR system. GAPDH served as the internal control for normalizing mRNA expressions. Relative expression levels were calculated using the 2^−ΔΔCT^ or 2^−ΔCT^ method. Primer sequences are listed in Table S2. The primers used in this study were synthesized by Applied Biosystems, Foster City, CA, USA.

### 2.4. Western Blot Analysis

Cells were harvested and lysed in RIPA buffer supplemented with protease inhibitor, phosphatase inhibitor and PMSF. Lysates were sonicated for 30 s and centrifuged at 14,000× *g* for 15 min. Supernatants were mixed with 5 × loading buffer and boiled at 95 °C for 10 min. Proteins were separated by SDS-PAGE, transferred to membranes, blocked for 15 min and incubated with primary antibodies overnight at 4 °C. After secondary antibody incubation, membranes were washed and developed. Antibodies used are as follows: GAPDH (1:1000, Proteintech, Rosemont, IL, USA, 10494-1-AP), KIF21B (1:1000, Aviva Systems Biology, San Diego, CA, USA, ARP66385_P050), p62 (1:1000, Proteintech, 18420-1-AP), P-akt (1:1000, Proteintech, 66444-1-Ig), PTEN (1:1000, Proteintech, 60300-3-Ig), cleaved caspase-3 (1:1000, Proteintech, 19677-1-AP), Ki-67 (1:1000, Proteintech, 27309-1-AP), Cyclin D1 (1:1000, Proteintech, 60186-1-Ig), C-myc (1:1000, Proteintech, 10828-1-AP), Beta Catenin (1:1000, Proteintech, 1067-2-AP), and TRIM3 (1:1000, Proteintech, 28392-1-AP). Secondary antibodies were HRP-conjugated Goat Anti-Rabbit IgG (1:5000, Proteintech, SA00001-2) and HRP-conjugated Goat Anti-Mouse IgG (1:5000, Proteintech, SA00001-1). The quantitative data of Western blot images is presented in Supplementary Information.

### 2.5. Cell Culture and Subculture

The human T-ALL cell lines Jurkat and CCRF-CEM (ATCC) were cultured in RPMI-1640 medium. The human embryonic kidney cell line HEK293T was maintained in Dulbecco’s Modified Eagle Medium (DMEM). Both media were supplemented with 10% fetal bovine serum (FBS) and 1% penicillin-streptomycin, and all cells were incubated at 37 °C in a humidified atmosphere containing 5% CO_2_. Cells were passed every 3 days by centrifugation at 1000 rpm for 5 min, washed with PBS and reseeded at 1:4 ratio. All experiments used cells in logarithmic growth phase with viability >95%.

### 2.6. siRNA and Lentiviral Transfection

KIF21B-targeting siRNA (sh-KIF21B) and negative control (sh-NC) were designed and synthesized by (GenePharma, Suzhou, China), as well as TRIM3-targeting siRNA (si-TRIM3) and negative control (si-NC). Plasmids encoding GFP-tagged PTEN (PTEN), FLAG-tagged KIF21B (FLAG-KIF21B), HA-tagged ubiquitin (HA-Ub) and 6his-tagged TRIM3 (6his-TRIM3) were purchased from Corues Biotechnology, Boston, MA, USA. Cells were transfected using Lipofectamine 3000 (Invitrogen, Carlsbad, CA, USA) according to the manufacturer’s instructions. Medium was replaced 8 h post-transfection and cells were harvested 72 h later.

For lentiviral infection, KIF21B-overexpressing and empty vector lentiviruses were constructed by Obio Technology, Shanghai, China. Cells were infected in the presence of polybrene. After 24 h, the medium was refreshed. Selection with puromycin (Thermo Fisher, Waltham, MA, USA) began 72 h post-infection and stable pools were established after 2 weeks.

### 2.7. CCK-8 Assay

Cells were seeded in 96-well plates with three replicates per group, including blank controls. After adding 10 µL CCK-8 solution (Beyotime, Shanghai, China) per well, plates were incubated for 2.5 h. Absorbance at 450 nm was measured. Data was normalized to the 0 h time point to generate growth curves.

### 2.8. Apoptosis Assay by Flow Cytometry

Apoptosis was assessed using Annexin V-FITC/PI double staining (Beyotime, China). Cells were washed with cold PBS and resuspended in 1 × Binding Buffer. Then, 100 µL cell suspension was stained with 5 µL Annexin V-FITC and 5 µL PI for 15 min at room temperature in the dark. After adding 400 µL Binding Buffer, samples were analyzed within 1 h. Early apoptotic (Annexin V^+^/PI^−^) and late apoptotic (Annexin V^+^/PI^+^) populations were quantified.

### 2.9. Animal Experiments

NSG mice were irradiated with a sublethal dose and injected intravenously with human Jurkat cells. Body weight and health were monitored regularly. Peripheral blood was analyzed by flow cytometry for hCD3^+^ leukemia cells. After 4 weeks, the mice were euthanized. Single-cell suspensions from the lymph node were analyzed by flow cytometry. The spleen and liver were weighed.

### 2.10. Multiplex Immunofluorescence (IF) Staining with Tyramide Signal Amplification (TSA)

Multiplex IF staining was performed on formalin-fixed, paraffin-embedded (FFPE) lymph node sections using the TSA method. Briefly, after routine deparaffinization and antigen retrieval, sections were blocked and incubated overnight at 4 °C with an anti-CD3 primary antibody (Proteintech, 17617-1-AP). Following washes, a horseradish peroxidase (HRP)-conjugated secondary antibody was applied. CD3 signal was developed using a Cy3-conjugated tyramide reagent, yielding a yellow-red fluorescence. Nuclei were subsequently stained with DAPI. For image analysis, the percentage of CD3-positive cells (Cy3 signal) relative to the total number of nuclei (DAPI signal) was quantified using ImageJ 2 software to determine the infiltration rate.

### 2.11. Immunohistochemistry (IHC) Staining and H-Score Analysis

Tumor tissues from the mice were fixed with 10% formalin, embedded in paraffin and cut into 5 µm sections. All specimens were evaluated for cleaved caspase-3, p62 and Ki-67 expression. The sections were photographed via a microscope. Whole sections were scanned and evaluated by two independent pathologists. Staining intensity was graded as 0 (negative), 1+ (weak), 2+ (moderate), or 3+ (strong). H-score was calculated using the formula: H-score = (1 × %1+) + (2 × %2+) + (3 × %3+), ranging from 0 to 300. Inter-rater reliability was assessed by intraclass correlation coefficient (ICC > 0.85). Individual IHC scoring data in liver and spleen tissues is presented in Table S3.

### 2.12. IF

Cells were fixed with 4% PFA, permeabilized with 0.5% Triton X-100, and blocked with 5% BSA. Primary antibodies against α-tubulin, KIF21Band TRIM3 were applied overnight at 4 °C. After washing, cells were incubated with Alexa Fluor 488- or 594-conjugated secondary antibodies (Beyotime, China) for 1 h. Nuclei were counterstained with DAPI (Beyotime, China). Images were acquired using a confocal microscope.

### 2.13. Bioinformatic Analysis

The STRING database (v11.5) was used to construct a protein–protein interaction network for KIF21B with high confidence (score > 0.7). Correlation between KIF21B and TRIM3 expression was analyzed via the Home for Researchers platform (www.home-for-researchers.com) using TCGA or CCLE datasets.

### 2.14. Co-Immunoprecipitation (Co-IP)

Cell lysates from Jurkat and CCRF-CEM cells were incubated with anti-KIF21B antibody or control IgG overnight at 4 °C. Protein A/G beads were added to capture complexes. After washing, bound proteins were eluted and detected by Western blot using anti-TRIM3 and anti-KIF21B antibodies.

### 2.15. Ubiquitination Assay

HEK293T cells were co-transfected with FLAG-KIF21B, HA-Ub and 6his-TRIM3 (experimental group) or empty vector (control). After 24 h, MG132 (MCE, Monmouth Junction, NJ, USA) was applied for 6 h. Cells were lysed and FLAG-tagged proteins were immunoprecipitated. Ubiquitination was detected with anti-HA antibody. Expression was verified with anti-FLAG, anti-6his and anti-GAPDH antibodies.

### 2.16. Protein Stability Assay

Cells transfected with si-NC or si-TRIM3 were treated with cycloheximide (CHX, 100 µg/mL) (MCE, USA) and harvested at 0, 2, 4, 6, 9 and 12 h. KIF21B levels were assessed by Western blot and degradation curves were plotted to compare half-lives.

### 2.17. Statistical Analysis

Data are presented as mean ± SD from at least three independent experiments. Statistical analyses were performed using GraphPad Prism 9.0. Comparisons between the two groups used Student’s t-test; multiple comparisons used one-way ANOVA with Tukey’s post hoc test. Survival curves were analyzed by the Kaplan–Meier method with log-rank test. *p* < 0.05 was considered significant (* *p* < 0.05, ** *p* < 0.01, *** *p* < 0.001).

## 3. Results

### 3.1. KIF21B Is Highly Expressed in T-ALL and Predicts Poor Prognosis

**Figure 1:**
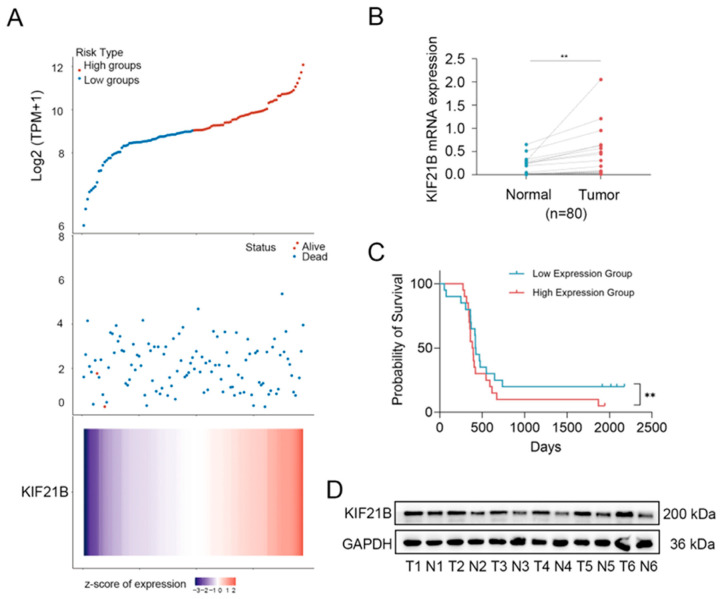
Clinical significance and expression validation of KIF21B in T-ALL. (**A**) We downloaded STAR-counts data and corresponding clinical information for pediatric T-ALL from the TARGET database. **Top**: scatter plot of gene expression levels (low to high) with color-coded groups; **middle**: distribution of survival time and status corresponding to gene expression; **bottom**: heatmap of gene expression. (**B**) qRT-PCR (*n* = 80) analysis of KIF21B expression in PBMCs from T-ALL patients (*n* = 40) and healthy donors (*n* = 40). (**C**) Kaplan–Meier survival analysis of T-ALL patients from (**B**) stratified by high or low KIF21B expression. (**D**) Western blot (*n* = 6) analysis of KIF21B expression in PBMCs from T-ALL patients and healthy donors. ** *p* < 0.01.

To investigate the clinical significance of KIF21B in T-ALL, we analyzed its expression and association with patient prognosis and diagnostic value. Patients were divided into high- and low-risk groups based on the median expression level. Those in the high-risk group exhibited significantly shorter overall survival (Figure 1A, Upper). A scatter plot of survival status and time further indicated a higher proportion of KIF21B high-expressing cases among deceased patients (Figure 1A, Middle). A heatmap illustrated the expression levels of KIF21B across different samples (Figure 1A, Lower). qRT-PCR confirmed that KIF21B mRNA expression was significantly upregulated in T-ALL patients compared with normal peripheral blood mononuclear cells (PBMCs) (Figure 1B). To assess the prognostic value of KIF21B, we stratified the T-ALL patient cohort (*n* = 40) based on its expression levels. Kaplan–Meier survival analysis revealed that patients with high KIF21B expression had a significantly poorer overall survival compared to those with low expression (Figure 1C). To further validate KIF21B expression in T-ALL patients, we performed Western blot using clinical samples. Western blot analysis consistently demonstrated increased protein expression of KIF21B (Figure 1D and Figure S1).

### 3.2. KIF21B Promotes Malignant Progression of T-ALL In Vitro and In Vivo

**Figure 2:**
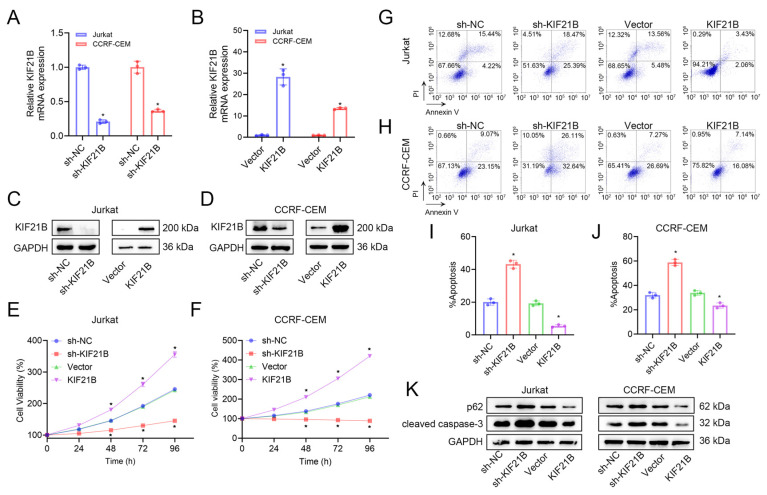
Effects of KIF21B knockdown and overexpression on T-ALL cell proliferation and apoptosis. (**A**,**B**) qRT-PCR and (**C**,**D**) Western blot analysis of transfection efficiency after lentivirus-mediated knockdown (sh-KIF21B) and overexpression (KIF21B) in Jurkat and CCRF-CEM cells. GAPDH was used as a loading control. (**E**,**F**) CCK-8 assay evaluating the effect of KIF21B knockdown and overexpression on cell proliferation. (**G**,**H**) Representative flow cytometry plots of Annexin V/PI staining. (**I**,**J**) Bar graphs showing apoptosis rates in different groups of the two cell lines. (**K**) Western blot analysis of apoptosis-related proteins p62 and cleaved caspase-3 after KIF21B knockdown or overexpression. GAPDH served as loading control. * *p* < 0.05.

We transfected Jurkat and CCRF-CEM cell lines with sh-KIF21B or KIF21B-overexpressing lentivirus. qRT-PCR (Figure 2A,B) and Western blot (Figure 2C,D and Figure S2) confirmed efficient knockdown and overexpression of both mRNA and protein levels, indicating successful cell model establishment. To explore the biological functions of KIF21B in T-ALL, we examined its effects on cell proliferation, apoptosis and tumorigenesis using in vitro and in vivo models. CCK-8 assays (Figure 2E,F) showed that KIF21B knockdown significantly inhibited cell proliferation, while its overexpression promoted proliferation. Flow cytometry revealed that KIF21B knockdown increased apoptosis, whereas overexpression suppressed it (Figure 2G–J). Western blot analysis further indicated that KIF21B knockdown upregulated the expression of apoptosis-related proteins cleaved caspase-3 and p62 (Figure 2K and Figure S3). To determine whether the oncogenic function of KIF21B depends on PTEN deficiency, we reconstituted wild-type PTEN in PTEN-aberrant T-ALL cells. Notably, the potent pro-proliferative effect driven by KIF21B overexpression persisted even upon PTEN restoration (Figure S4). These results indicated that the oncogenic role of KIF21B operates independently of PTEN status.

**Figure 3:**
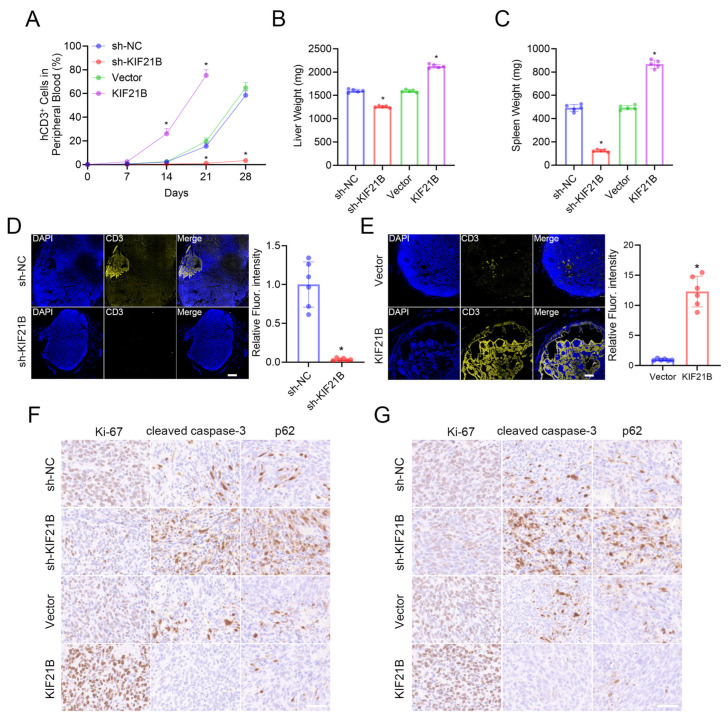
KIF21B-promoted leukemogenesis in a T-ALL xenograft model. (**A**) Dynamic monitoring of human CD3^+^ leukemia cell proportion in peripheral blood of mice by flow cytometry (*n* = 5). (**B**) Spleen and (**C**) liver weights of mice from the indicated groups (*n* = 5). (**D**,**E**) Representative multi-color IF images of lymph node sections stained for CD3 and their quantitative data analysis. Scale bar, 500 μm. (**F**,**G**) Representative IHC images of liver (**F**) and spleen (**G**) sections from mice subjected to four different treatments: sh-NC, sh-KIF21B, Vector and KIF21B. Tissue sections were stained for Ki-67, cleaved caspase-3 and p62. Scale bar, 50 µm (*n* = 5). * *p* < 0.05.

In vivo, the leukemogenic capacity of KIF21B was assessed in a xenograft mouse model. Longitudinal flow cytometric analysis of peripheral blood revealed that KIF21B knockdown significantly decreased the proportion of human CD3^+^ leukemia cells over time, whereas KIF21B overexpression accelerated their expansion, compared to respective controls. Consistent with this, endpoint analysis demonstrated a significant reduction in human CD3^+^ cell infiltration within the axillary lymph nodes of the knockdown group (Figure 3A). The splenomegaly and hepatomegaly characteristics of advanced leukemia were markedly attenuated in KIF21B-knockdown mice, as evidenced by reduced organ weights, whereas KIF21B overexpression exacerbated these pathological changes (Figure 3B,C). Furthermore, multiplex IF staining of lymph node sections visually confirmed a lower burden of CD3^+^ leukemic cells upon KIF21B knockdown, an effect that was quantitatively supported by fluorescence intensity analysis (Figure 3D,E). IHC analysis (Figure 3F,G and Figure S5) revealed that the knockdown of KIF21B significantly upregulated the expression of cleaved caspase-3 and p62 while paradoxically also decreasing Ki-67. These effects were reversed in the KIF21B-overexpressing group. Collectively, these results substantiated that KIF21B potently promoted T-ALL progression and impaired autophagic flux in vivo.

### 3.3. KIF21B Promotes Leukemia Progression by Regulating the Wnt/β-Catenin Signaling Pathway

**Figure 4:**
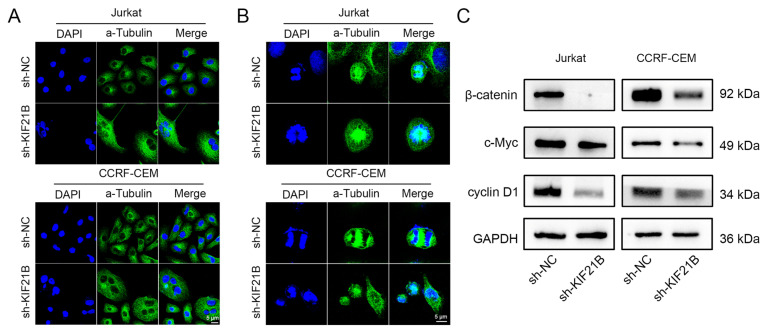
KIF21B-regulated mitotic spindle assembly and Wnt/β-catenin signaling. (**A**,**B**) IF images of mitotic spindles (**A**) and chromosome distribution (**B**) in Jurkat and CCRF-CEM cells. Green: α-Tubulin; blue: DAPI. Scale bar: 5 μm. (**C**) Western blot analysis of β-catenin phosphorylation and downstream targets (c-Myc and cyclin D1) after KIF21B knockdown.

To investigate the potential molecular mechanisms by which KIF21B influences the malignant phenotype of T-ALL, we examined its effects on microtubule structure and the Wnt/β-catenin signaling pathway. IF staining revealed significant abnormalities in microtubule structure in KIF21B-knockdown Jurkat and CCRF-CEM cells compared with controls. Control cells displayed well-organized microtubule fibers and typical bipolar spindles, whereas KIF21B-knockdown cells showed disordered spindle structures, unorganized microtubule arrays and abnormal lengths (Figure 4A). Chromosome alignment was also impaired, with most cells arrested in prometaphase exhibiting scattered chromosomes that failed to congress properly (Figure 4B). These results suggest that KIF21B loss leads to defects in spindle assembly and chromosome segregation during mitosis, highlighting its key role in maintaining normal mitotic progression in leukemia cells. Western blot analysis showed that KIF21B knockdown significantly reduced the β-catenin and downregulated the expression of c-Myc and cyclin D1 in both cell lines (Figure 4C and Figure S6).

### 3.4. KIF21B Interacts with TRIM3 and Co-Localizes in the Cytoplasm

**Figure 5:**
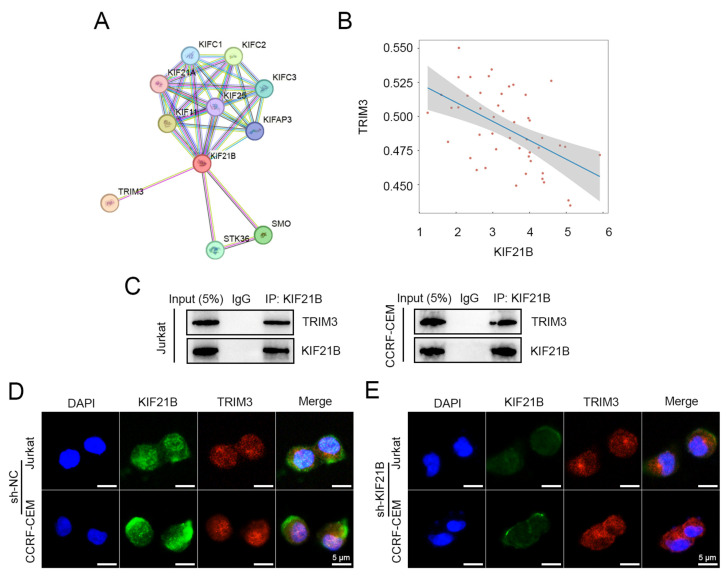
Interaction and co-localization of KIF21B and TRIM3. (**A**) Protein–protein interaction network of KIF21B constructed using STRING database. (**B**) Correlation analysis between KIF21B and TRIM3 gene expression in TCGA/CCLE datasets via Home for Researchers platform. (**C**) Co-immunoprecipitation assay using anti-KIF21B antibody in Jurkat and CCRF-CEM cell lysates. IgG served as control. Interaction was detected by Western blot. (**D**,**E**) Subcellular localization of KIF21B (green) and TRIM3 (red) by IF in cells transfected with sh-KIF21B and its negative control. Images were acquired under identical confocal fluorescence microscopy parameters. Nuclei were stained with DAPI (blue). Scale bar: 5 μm.

Protein–protein interaction network analysis based on the STRING database indicated that KIF21B may interact with several Kinesin family members and TRIM3 (Figure 5A). Correlation analysis further revealed a significant negative correlation between KIF21B and TRIM3 expression (Figure 5B). To validate their interaction, co-immunoprecipitation was performed. Results showed that the KIF21B antibody effectively enriched TRIM3 protein in both Jurkat and CCRF-CEM cells, while the IgG control did not, confirming a specific binding between KIF21B and TRIM3 (Figure 5C and Figure S7). IF staining demonstrated that KIF21B and TRIM3 widely co-localized in the cytoplasm, suggesting potential functional cooperation within cells (Figure 5D). Moreover, in cells transfected with sh-KIF21B, the green signal for KIF21B was diminished while the red signal for TRIM3 remained unchanged (Figure 5E).

### 3.5. TRIM3 Modulates T-ALL Cell Proliferation by Promoting Ubiquitination-Dependent Degradation of KIF21B and Regulating the Wnt/β-Catenin Pathway

**Figure 6:**
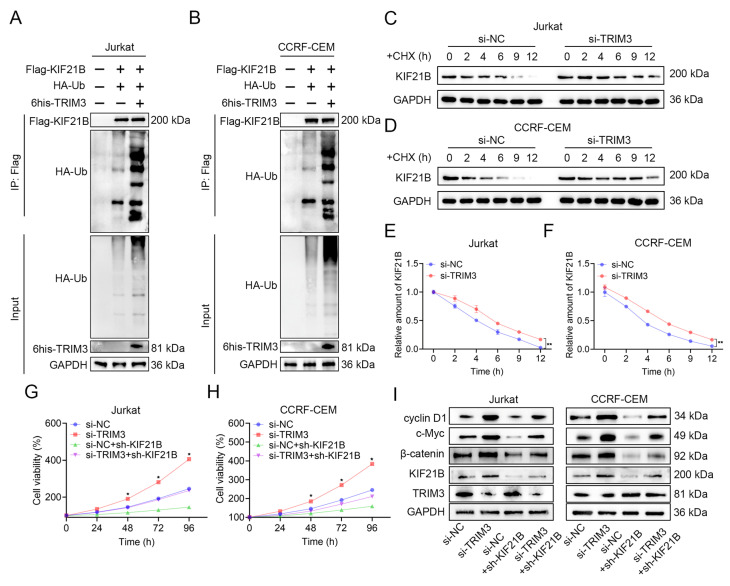
Mechanistic studies on TRIM3-mediated regulation of KIF21B. (**A**,**B**) Ubiquitination assay in Jurkat (**A**) and CCRF-CEM cells (**B**) co-transfected with FLAG-KIF21B, HA-Ub and 6his-TRIM3, treated with MG132 (10 μM, 6h). Immunoprecipitation was performed with anti-FLAG antibody. (**C**,**D**) CHX chase assay (100 μg/mL) in cells transfected with si-NC or si-TRIM3. KIF21B degradation was analyzed by Western blot at indicated time points. (**E**,**F**) Quantification of KIF21B protein levels using ImageJ. (**G**,**H**) CCK-8 assay showing proliferation curves of different treatment groups (si-NC, si-TRIM3, sh-KIF21B and double knockdown) over 96 h. (**I**) Western blot analysis of KIF21B, TRIM3, β-catenin, cyclin D1and c-Myc in different treatment groups. * *p* < 0.05, ** *p* < 0.01.

Ubiquitination assays showed that TRIM3 overexpression significantly enhanced ubiquitination of KIF21B (Figure 6A,B and Figures S8 and S9). Knockdown of TRIM3 in Jurkat and CCRF-CEM cells increased stability and extended the half-life of KIF21B protein (Figure 6C–F and Figure S10). Functionally, the proliferation rate of the dual-knockdown group was significantly lower than that of the TRIM3 single-knockdown group, indicating that concurrent KIF21B knockdown partially rescues the pro-proliferative effect caused by TRIM3 loss (Figure 6G,H). Rescue experiments demonstrated that dual knockdown of TRIM3 and KIF21B partially reversed the upregulation of β-catenin, cyclin D1 and c-Myc protein levels induced by TRIM3 knockdown alone (Figure 6I and Figure S11).

## 4. Discussion

**Figure 7:**
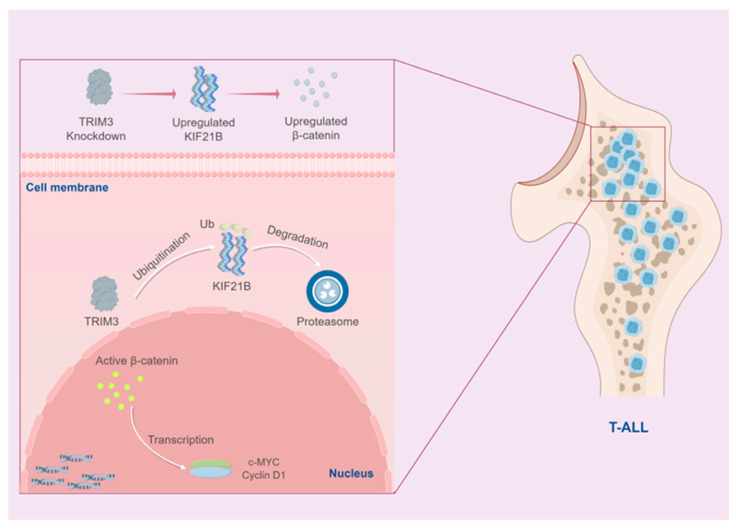
Schematic illustration of the mechanism by which TRIM3 inhibits the Wnt/β-catenin signaling pathway through ubiquitin-mediated degradation of KIF21B. In T-ALL, TRIM3 mediates the ubiquitination and degradation of KIF21B, thereby negatively regulating its protein stability. Upregulation of KIF21B promotes the phosphorylation of β-catenin and activates the Wnt/β-catenin signaling pathway, leading to the enhanced expression of c-Myc and cyclin D1.

Through an integrated approach combining bioinformatic analysis, in vitro functional experiments and clinical sample validation, this study systematically unravels the oncogenic role and molecular mechanisms of KIF21B in T-ALL for the first time. We demonstrated that KIF21B is significantly overexpressed in T-ALL and closely associated with poor patient prognosis. Functionally, it drives tumor cell proliferation and suppresses apoptosis by activating the Wnt/β-catenin signaling pathway. More importantly, we identified the E3 ubiquitin ligase TRIM3 as an upstream negative regulator of KIF21B, which promotes its degradation via the ubiquitin-proteasome pathway, thereby defining a novel TRIM3–KIF21B–β-catenin regulatory axis critical for T-ALL malignant progression (Figure 7).

One of the most significant findings of this work is the potential of KIF21B as a biomarker for poor prognosis in T-ALL. Its expression level was significantly correlated with shorter overall survival and exhibited high predictive value for 5-year survival rates, offering a new potential molecular indicator for risk stratification in T-ALL. At the mechanistic level, our results link the function of KIF21B to the canonical Wnt/β-catenin signaling pathway. Knockdown of KIF21B led to marked downregulation of β-catenin, c-Myc and cyclin D1 protein levels, consistent with the well-established role of this pathway in proliferation and inhibiting apoptosis. Notably, KIF21B is a microtubule-associated motor protein [14,21]. Our findings that its depletion causes severe spindle assembly and chromosome alignment defects suggest that KIF21B may maintain mitotic fidelity, thereby providing a permissive cellular environment for the stable accumulation of key oncoproteins like β-catenin. This discovery provides strong new evidence supporting the concept of functional crosstalk between cytoskeletal dynamics and oncogenic signal transduction [22,23], broadening our understanding of T-ALL pathogenesis.

The most salient highlight of this study lies in elucidating the upstream regulatory mechanism controlling KIF21B protein stability. Through multiple experimental approaches, we confirmed that TRIM3 interacts with KIF21B and mediates its ubiquitination and degradation. This finding carries substantial scientific importance, as TRIM family proteins often play dual roles in cancer [19,24,25,26,27]. Our research suggests that TRIM3 may act as a tumor suppressor in T-ALL, whereby its reduced expression (or loss of function) leads to an accumulation of KIF21B protein, subsequently activating downstream oncogenic pathways. The delineation of this TRIM3–KIF21B regulatory relationship provides a mechanistic explanation for the aberrant overexpression of KIF21B in tumors.

Our study elucidates a novel role for the TRIM3–KIF21B regulatory axis in cancer. In contrast to the function reported by Labonté et al. [20], wherein TRIM3 modulates the motility of KIF21B in neurons, we demonstrate that in T-ALL cells, TRIM3 acts as an E3 ubiquitin ligase mediating the ubiquitin-dependent degradation of KIF21B. This fundamental difference in function may stem from cell-type specificity or distinct disease contexts. In cancer cells, aberrantly high expression of KIF21B likely drives oncogenic signaling and its negative regulation via TRIM3-mediated degradation may represent a mechanism to maintain cellular homeostasis. The discovery of this mechanism offers new perspectives for therapeutic strategies targeting KIF21B in cancer.

Our findings possess dual implications. On a theoretical level, they reveal a novel post-translational regulatory mechanism mediated by TRIM3 and identify KIF21B as a new upstream regulator of the Wnt pathway, enriching the molecular regulatory network of T-ALL. In terms of clinical translation, KIF21B serves as both an independent prognostic factor and a promising therapeutic target. Developing small-molecule inhibitors or PROTAC degraders targeting KIF21B itself or the TRIM3–KIF21B interaction interface could offer a novel precision therapy strategy for T-ALL patients with high KIF21B expression and poor prognosis.

Nevertheless, this study has certain limitations. First, the direct molecular mechanism by which KIF21B regulates β-catenin phosphorylation is not fully elucidated; whether it interacts with other scaffolding proteins or kinase complexes warrants further investigation. Second, the current functional studies primarily relied on cell lines; future validation using patient-derived xenograft (PDX) models would better recapitulate the clinical heterogeneity of T-ALL. Finally, besides TRIM3, whether other transcriptional factors or epigenetic mechanisms regulate KIF21B transcription remains an open question.

## 5. Conclusions

Our work establishes the role of KIF21B as a critical oncogene in T-ALL and elucidates a novel mechanism for its regulation via TRIM3-mediated ubiquitination. Future studies should focus on resolving the structure of the KIF21B–TRIM3 complex to guide drug design and explore the synergistic effects of targeting this axis in combination with conventional chemotherapy or other targeted agents, ultimately aiming to provide new strategies for improving the clinical outcomes of T-ALL patients.

## Data Availability

Data will be made available on request.

## Supplementary Materials

The following supporting information can be downloaded at: https://www.mdpi.com/article/10.3390/cancers18091327/s1, Figure S1. Original western blots corresponding to Figure 1D. Figure S2. Original western blots corresponding to Figure 2C,D. Figure S3. Original western blots corresponding to Figure 2K. Figure S4. Analysis of PTEN reconstitution in PTEN-aberrant T-ALL cells. Figure S5. H-score quantification of Ki-67, Cleaved Casepase 3 and p62 expression in liver (A–C) and spleen (D–F) from the indicated groups (n = 5 per group). H-score was calculated as Σ (percentage of positive cells × staining intensity, 0–3). Data were shown as mean ± SD. Figure S6. Original western blots corresponding to Figure 4C. Figure S7. Original western blots corresponding to Figure 5C. Figure S8. Ubiquitination assay in HEK293T cells. Figure S9. Original western blots corresponding to Figure 6A,B. Figure S10. Original western blots corresponding to Figure 6C,D. Figure S11. Original western blots corresponding to Figure 6I. Table S1. Clinical and Molecular Characteristics of T-ALL Patients (n = 40). Table S2. The primers used in this study. Table S3. Individual IHC scoring data in liver and spleen tissues.
