# Supplementary material for: KIF21B, Ubiquitinated by TRIM3, Exerts Oncogenic Role in T-Cell Acute Lymphoblastic Leukemia by Activating Wnt/β-Catenin Pathway
Source: Cancers (Basel). 2026 Apr 22;18(9):1327. doi: 10.3390/cancers18091327 (PMC13162888; doi:10.3390/cancers18091327)
Supplement: Supplementary file 1 [file cancers-18-01327-s001.zip › Supplementary Information.pdf]

Supplementary Information

KIF21B, Ubiquitinated by TRIM3, Exerts Oncogenic Role in T-Cell Acute Lymphoblastic Leukemia by Activating Wnt/β-catenin Pathway

Yu Sun[a], Yuhao Xu[a] and Chao Lu[a,*]

[a] Department of pediatrics, the First Affiliated Hospital with Nanjing Medical University, Nanjing, Jiangsu, 210029, China.

[*] Corresponding author. Department of pediatrics, the First Affiliated Hospital with Nanjing Medical University, Nanjing, Jiangsu210029, China.

E-mail addresses:

Yu Sun [a]: victmsun@163.com

Yuhao Xu[a]: 15250962215@163.com

Chao Lu [a,*]: luchao_doctor@163.com.

Department of pediatrics, the First Affiliated Hospital with Nanjing Medical University, Nanjing, Jiangsu210029, China

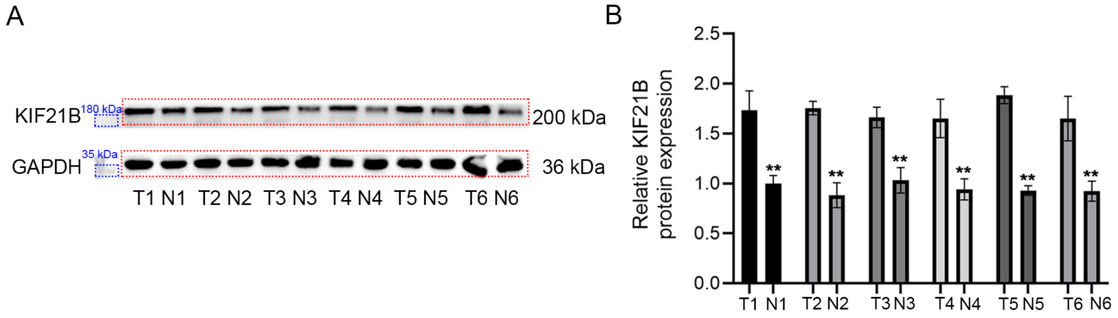

Figure S1. Original western blots corresponding to Figure 1D. (A) Blue boxes: molecular weight markers. Red boxes: bands cropped for the main figure. Molecular sizes (kDa) were indicated. **(B)** Quantification data in Figure 1D.

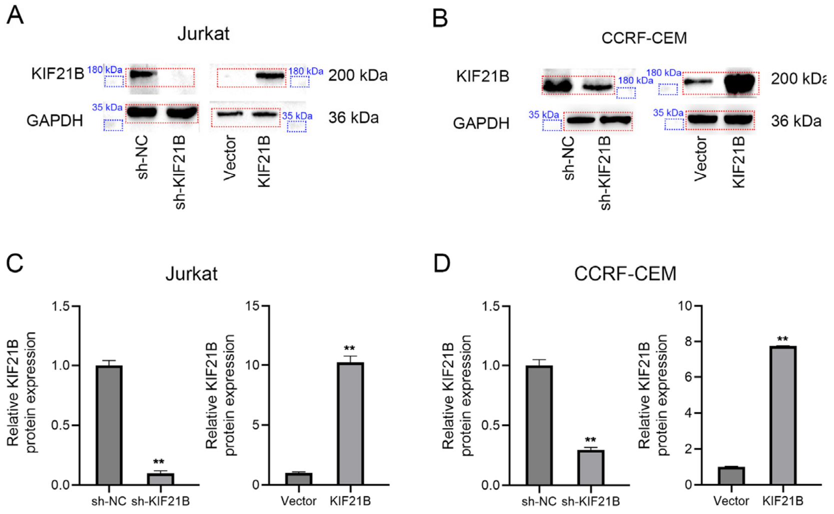

Figure S2. Original western blots corresponding to Figure 2C,D. (A-B) Blue boxes: molecular weight markers. Red boxes: bands cropped for the main figure. Molecular sizes (kDa) were indicated. **(C-D)** Quantification data in Figure 2C-D.

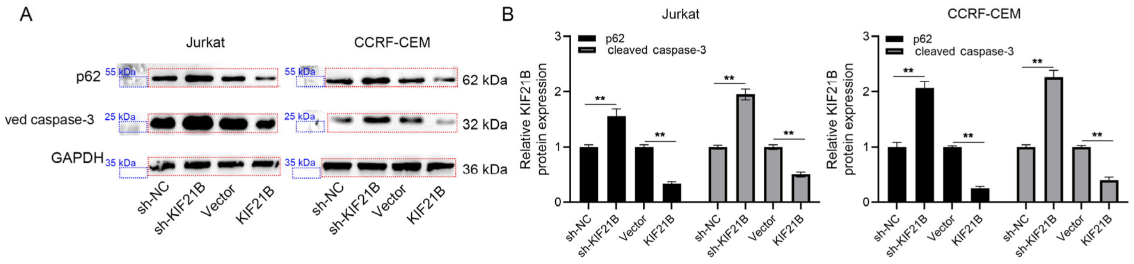

Figure S3. Original western blots corresponding to Figure 2K. (A) Blue boxes: molecular weight markers. Red boxes: bands cropped for the main figure. Molecular sizes (kDa) were indicated. **(B)** Quantification data in Figure 2K.

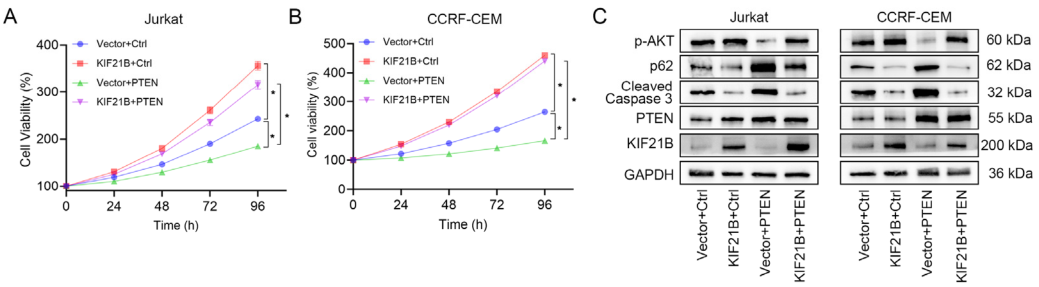

Figure S4. Analysis of PTEN reconstitution in PTEN-aberrant T-ALL cells. (A-B) CCK-8 cell proliferation assays in (A) Jurkat and (B) CCRF-CEM cells co-transfected with the indicated plasmids (Vector or KIF21B overexpression, together with control or wild-type PTEN plasmid). (C) Western blot analysis under different treatment conditions.

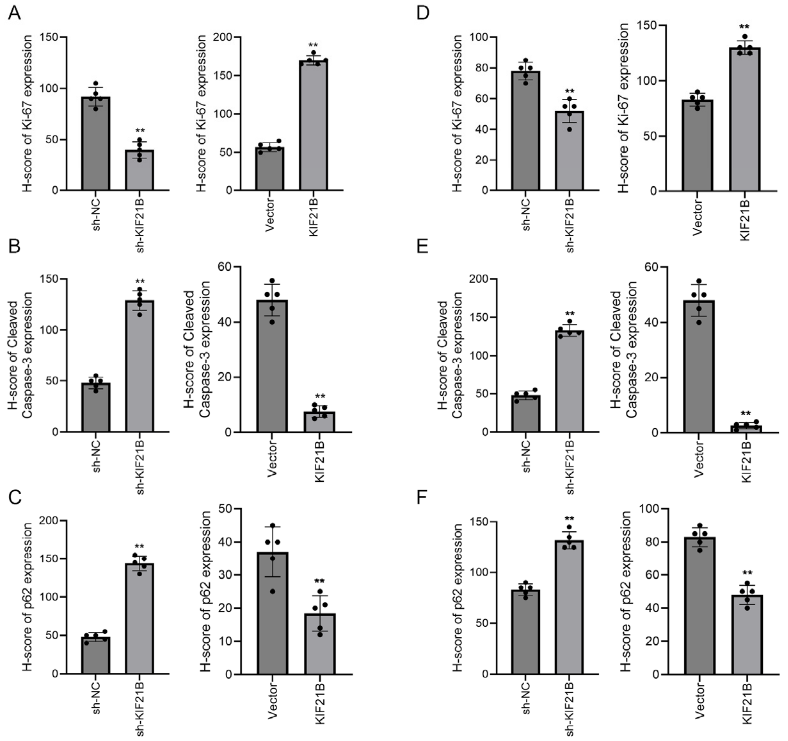

Figure S5. H-score quantification of Ki-67, Cleaved Casepase 3 and p62 expression in liver (A-C) and spleen (D-F) from the indicated groups (n=5 per group). H-score was calculated as Σ (percentage of positive cells × staining intensity, 0–3). Data were shown as mean ± SD.

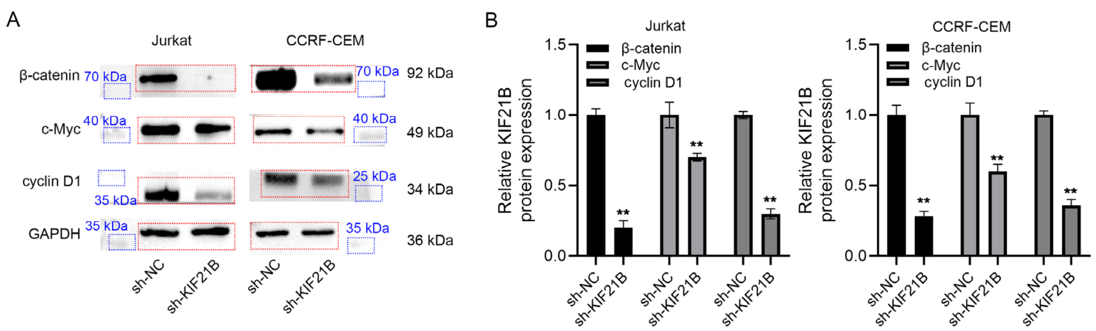

Figure S6. Original western blots corresponding to Figure 4C. (A) Blue boxes: molecular weight markers. Red boxes: bands cropped for the main figure. Molecular sizes (kDa) were indicated. **(B)** Quantification data in Figure 4C.

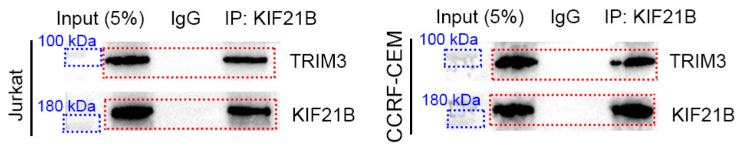

Figure S7. Original western blots corresponding to Figure 5C. Blue boxes: molecular weight markers. Red boxes: bands cropped for the main figure. Molecular sizes (kDa) were indicated.

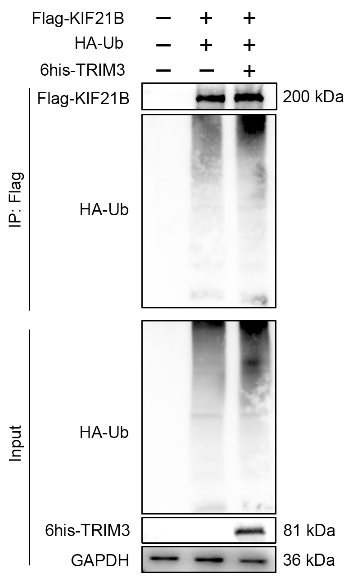

Figure S8. Ubiquitination assay in HEK293T cells.

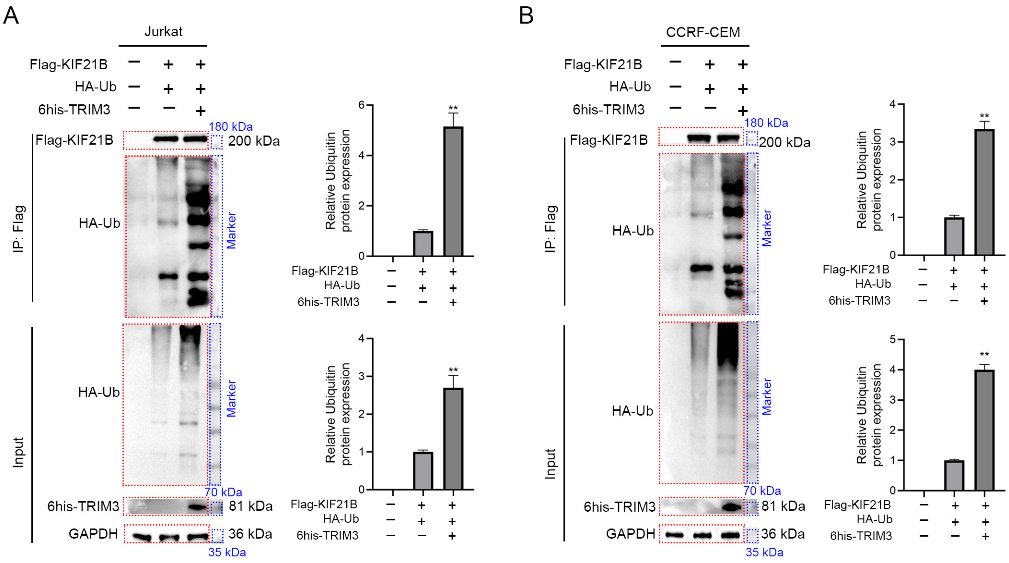

Figure S9. Original western blots corresponding to Figure 6A,B. Blue boxes: molecular weight markers. Red boxes: bands cropped for the main figure. Molecular sizes (kDa) were indicated. Quantification data were shown next to the bands.

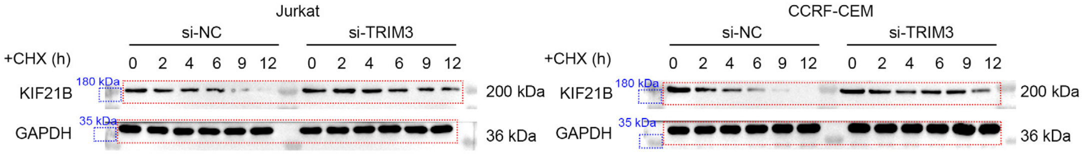

Figure S10. Original western blots corresponding to Figure 6C,D. Blue boxes: molecular weight markers. Red boxes: bands cropped for the main figure. Molecular sizes (kDa) were indicated.

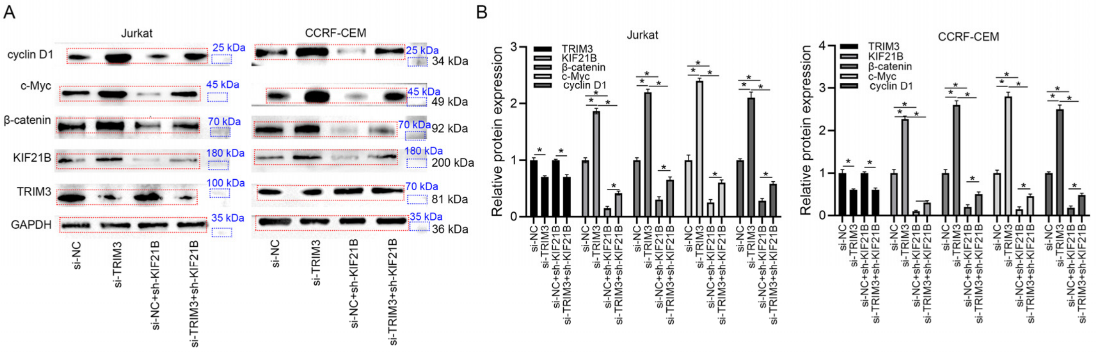

Figure S11. Original western blots corresponding to Figure 6I. (A) Blue boxes: molecular weight markers. Red boxes: bands cropped for the main figure. Molecular sizes (kDa) were indicated. **(B)** Quantification data in Figure 6I.
