# Supplementary material for: KIF21B, Ubiquitinated by TRIM3, Exerts Oncogenic Role in T-Cell Acute Lymphoblastic Leukemia by Activating Wnt/β-Catenin Pathway
Source: Cancers (Basel). 2026 Apr 22;18(9):1327. doi: 10.3390/cancers18091327 (PMC13162888; doi:10.3390/cancers18091327)
Supplement: Supplementary file 1 [file cancers-18-01327-s001.zip › Table S1.pdf]

Table S1. Clinical and Molecular Characteristics of T-ALL Patients (n=40)

**Characteristic**	**Value**
**Demographics**	
Age at diagnosis, years	
Median (Range)	14 (3 - 42)
< 18 years (Pediatric), n (%)	28 (70%)
≥ 18 years (Adult), n (%)	12 (30%)
Gender, n (%)	
Male	32 (80%)
Female	8 (20%)
**Disease Baseline**	
White Blood Cell (WBC) count, $\times 10^9$/L	
Median (Range)	92.0 (10.5 - 480.0)
WBC ≥ 100 $\times 10^9$/L (High), n (%)	18 (45%)
Immunophenotype (by flow cytometry)	T-ALL (100%)
**T-ALL Subtype (Early T-cell Precursor, ETP), n (%)**	10 (25%)
**Risk Stratification**	
Pediatric (NCI Criteria for T-ALL)*	(n=28)
Standard Risk (WBC < 100$\times 10^9$/L)	16 (57%)
High Risk (WBC ≥ 100$\times 10^9$/L)	12 (43%)
Adult (ECOG Performance Status 0-1), n (%)	12 (100%)
